# Postulation of Specific Disease Resistance Genes in Cereals: A Widely Used Method and Its Detailed Description

**DOI:** 10.3390/pathogens11030284

**Published:** 2022-02-23

**Authors:** Antonín Dreiseitl

**Affiliations:** Department of Integrated Plant Protection, Agrotest Fyto Ltd., 76701 Kroměříž, Czech Republic; dreiseitl@vukrom.cz

**Keywords:** barley powdery mildew, *Blumeria graminis* f. sp. *hordei*, *Hordeum vulgare*, cereals, biotrophic pathogens, resistance gene postulation

## Abstract

Cultivation of resistant varieties is an environmentally friendly and inexpensive method of crop protection. Numerous alleles of specific disease resistance occur in cereals and other crops, and knowledge of their presence in individual varieties has wide utilization in research and practice. Postulation based on phenotyping host—pathogen interactions and the gene-for-gene model is a common way of identifying these genes. The same technique and design of tests are used for postulating virulence when pathogen populations are studied. Powdery mildews caused by different *formae speciales* of *Blumeria graminis* (*Bg*) are important cereal diseases. In this contribution, experimental methods are described that use a model organism *Bg* f. sp. *hordei,* which can be employed for other cereal mildews and possibly rusts. It includes illustrations and a summary of our long-term practical experience. It also critically evaluates the benefits of leaf segment tests compared with screening whole plants.

## 1. Introduction

Powdery mildews caused by different *formae speciales* of the airborne ascomycete fungus *Blumeria graminis* (DC.) E. O. Speer are important diseases of cereals (barley, wheat, rye, oat and triticale). Cultivation of resistant varieties is an environmentally friendly and cheap method of crop protection. Because of spontaneous mutations, genes of specific resistance against diseases have occurred randomly in wild relatives and landraces of crops and have been used for the directed breeding of resistant varieties.

The model organism in our laboratory is *Blumeria graminis* f. sp. *hordei* (*Bgh*), causing the most frequent disease of barley [1,2]. Numerous barley powdery mildew resistance alleles (*Ml* alleles) have been transferred into cultivated varieties [3] bred mainly in central and north-west Europe [4,5]. In addition, older varieties often contain completely ineffective genes with no positive effect on resistance in the field. Knowledge of the presence of these genes in individual varieties has wide utilization in research and practice, as described in a later section.

The most common ways to identify genes of specific resistance are: (i) genetic analysis of progenies after crossing [6] based on Mendel’s laws of inheritance, (ii) postulation, which is founded on the gene-for-gene model [7], (iii) a novel method combining both of these [8] and (iv) molecular markers [9].

Identifying genes of specific resistance by postulation is based on recording the phenotypic responses of a variety after inoculation with numerous pathogen isolates to obtain an infection response array (IRA). Comparing the IRA of a tested variety with IRAs of standard genotypes possessing known resistance genes can result in the postulation of known or unknown genes and their combinations [10,11]. As it is not unusual to find five or more resistance genes present in one genotype [12], postulation is the most efficient method to analyze such complex data compared with other techniques, including sequencing.

The primary importance of resistance genes is protecting barley against powdery mildew. However, their postulation has many other applications, including: (1) mapping the distribution of native resistance worldwide [13,14,15]; (2) the selection of genetic resources for crop breeding [16,17]; (3) the selection of well-characterized genotypes for evaluating partial resistance [18]; (4) assessing efficacy and expected durability of resistance of varieties including potential candidates for registration [19]; (5) description of new resistance in registered varieties [20]; (6) identifying varieties and confirming their authenticity and pedigrees [21]; (7) selection of differential varieties for studying pathogen populations [22] and their evolution [23]; (8) uncovering genotypic contamination [24]; and (9) basic research including the effect of *Ml* alleles on resistance to other pathogens [25,26].

The aim of this paper is to provide a detailed account of postulating barley powdery mildew resistance genes that have long been widely used in our laboratory. The following, slightly modified description of the method for postulating specific barley powdery mildew resistance genes (Materials and Methods), published in a recent paper [24], is provided as an example and a starting point and is supplemented with detailed notes and other relevant information.

## 2. Materials and Methods

### 2.1. Plant Material and Pathogen Isolates

All 172 accessions of the core collection of the Czech gene bank of winter barley were studied. The varieties originated from 35 countries, 80% of which were from Europe. The most frequent were those from Germany, including the former GDR, followed by the USA (13), the Czech Republic, including the former Czechoslovakia (12), France (11) and the Soviet Union (10 accessions).

For resistance tests, 51 reference isolates of *Bgh* were used, which had been collected in 11 countries in all nonpolar continents over a period of 63 years (1953–2016) and represent the global virulence/avirulence diversity of the pathogen. The responses of 35 barley genotypes carrying different genes of specific resistance to these isolates have been presented previously [12]. Before inoculation, all isolates were checked for their purity and their correct pathogenicity phenotypes were verified on standard barley lines [27]. The isolates were multiplied on leaf segments of the check susceptible cv. Stirling [15].

### 2.2. Testing Procedure

About 50 seeds of each accession were sown in two pots (80 mm diameter) filled with a gardening peat substrate and placed in a mildew-proof greenhouse under natural daylight. The primary leaves were excised when the second leaves were emerging, and leaf segments 15 mm long were cut from the middle part of healthy fully expanded leaves. Three segments of each accession were placed on the surface of the media (0.8% water agar containing 40 mg^−L^ of benzimidazole—A leaf senescence inhibitor) in a 150 mm Petri dish. Leaf segments were placed adjacent to each other along with four segments of Stirling oriented diagonally with their adaxial surfaces facing upward.

**Figure 1:**
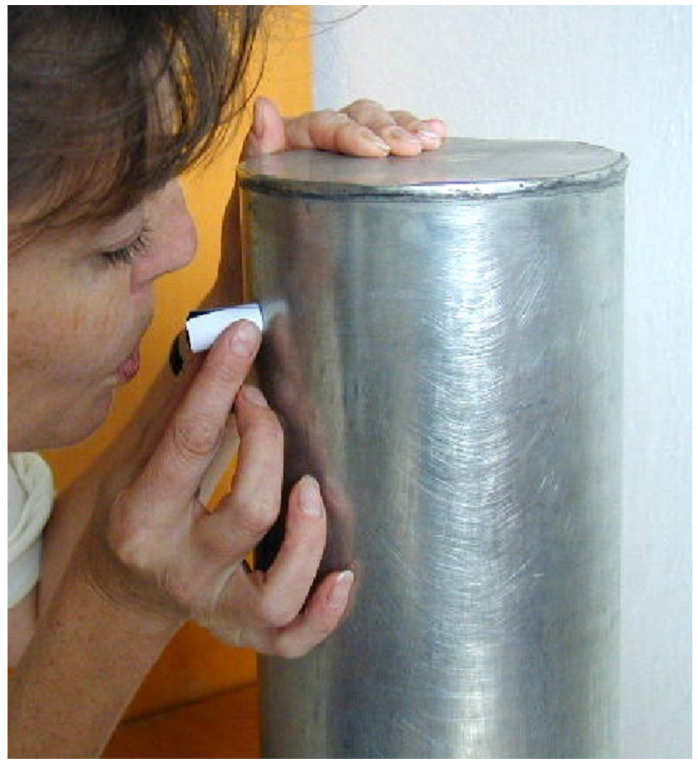
Inoculation—briskly blowing the inoculum into the inoculation tower. Petri dish with inoculated material is not visible, but is inside at the base of the tower.

For inoculation, a cylindrical metal settling tower of 150 mm diameter and 415 mm in height closed at the top was used, and a dish with segments was placed at the bottom of the tower. Conidia of each isolate taken from a leaf segment of the susceptible variety with fully-developed pathogen colonies were shaken onto a square piece (40 × 40 mm) of black paper to visually control the amount of inoculum deposited. Then, the paper was rolled to form a blowpipe and conidia of the isolate were blown through a side hole of 13 mm diameter with its center 50 mm from the upper end into the settling tower (Figure 1) over the Petri dish at a concentration of ca. 10 conidia mm^−2^. The dishes with inoculated leaf segments were incubated at 20 ± 1 °C under cool-white fluorescent lamps providing 12 h light at 30 ± 5 μmol m^−2^ s^−1^.

### 2.3. Evaluation

**Figure 2:**
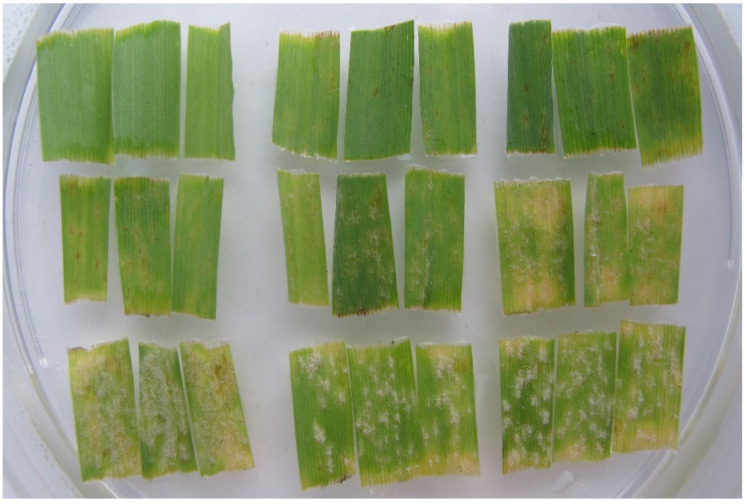
Nine infection responses developed seven days after inoculation with a powdery mildew isolate, each represented by three barley leaf segments that carry different resistance genes.

Seven days after inoculation, infection responses (IR = phenotype of accession x isolate interaction) that developed on the adaxial side of leaf segments were scored on a scale of 0–4 [28], where 0 = no mycelium and sporulation, and 4 = strong mycelial growth and sporulation (Table 1, Figure 2). IRs 3, 3–4 and 4 were considered susceptible. Each accession was tested with a minimum of two replications. If there were significant differences in IRs between replicates, additional tests were done. A set of 51 IRs provided an infection response array (IRA) for each accession. Based on the gene-for-gene hypothesis [7], the resistance genes in accessions were postulated by comparing their IRAs with previously determined IRAs of standard barley genotypes possessing known resistance genes. Generally, IRs 0 to 2–3 were considered resistant, but a typical IR for each resistance gene was also taken into account, e.g., *Mla13* has a typical IR 0, but if IR 2–3 was found, then it was considered as a susceptible response to this gene.

## 3. Detailed Remarks

### 3.1. Preparation of Material, Host Varieties and Isolates of the Pathogen

For water agar, a solution is prepared in advance by putting 0.8 g of benzimidazole into 1 L of distilled water and shaking well. To prepare the agar medium itself, 50 mL of the solution is added to 950 mL of distilled water to give the desired concentration (40 ppm). The required amount (8 g L^−1^) of finely ground agar is put into the solution and mixed with a glass rod. After a short boiling of about 3 min, the water agar is mixed again, allowed to cool to about 50 to 60 °C, poured into Petri dishes with a diameter of 150 mm in an amount of 50 mL per dish and the dishes are closed with lids.

From the first leaves of the tested or susceptible control barley variety, segments are excised and placed in a Petri dish 90 mm in diameter. From the appropriate number of 150 mm Petri dishes containing solidified agar (usually around 30 dishes), lids are removed and four of them placed on top of each other. The dishes are taken one at a time and the appropriate number (usually one triplet) of leaf segments of one accession is placed in each of them with tweezers. This is repeated with all varieties (usually 30 or 26) of the tested series. Subsequently, each dish with a set of segments is inoculated with one isolate, as described above. After briskly blowing the inoculum into the inoculation tower, it is necessary to wait about 30 s to settle the inoculum. This time allows the recording of the isolate designation and inoculation date on the lid of the Petri dish.

After each inoculation, the inside of the inoculation tower, the working surface and the tools used are sterilized with cotton wool moistened with 96% ethyl alcohol. The equipment comprises tweezers and a metal or plastic ring 30 mm high and 50 mm in diameter, which facilitates shaking the inoculum and prevents spores from falling off the black paper square. After sterilization of the interior of the inoculation tower, ethyl alcohol vapor is blown out and the tower is turned upside down to allow the remaining vapor to evaporate. In the meantime, another isolate can be prepared for inoculation. A fresh square of black paper is always used for inoculation with the next isolate, but can be further re-used without sterilization for about 24 h.

The inoculum of the isolates is prepared in advance. It is also multiplied on leaf segments of a susceptible variety, usually on Petri dishes with a diameter of 90 mm. Segments are inoculated at a low concentration of approx. 1 conidium mm^−2^. Inoculated dishes are stored in an illuminated refrigerator at 5 ± 1 °C under artificial light, cool-white fluorescent lamps providing 12 h light at about 10 μmol m^−2^ s^−1^, where they can be maintained for 6 to 8 weeks. If the inoculum is needed earlier than this, dishes are placed in the incubation room in advance. If isolates need to be prepared earlier still, they can be multiplied in the incubation room and used 10 to 14 days after inoculation (dai).

### 3.2. Test Design

**Figure 3:**
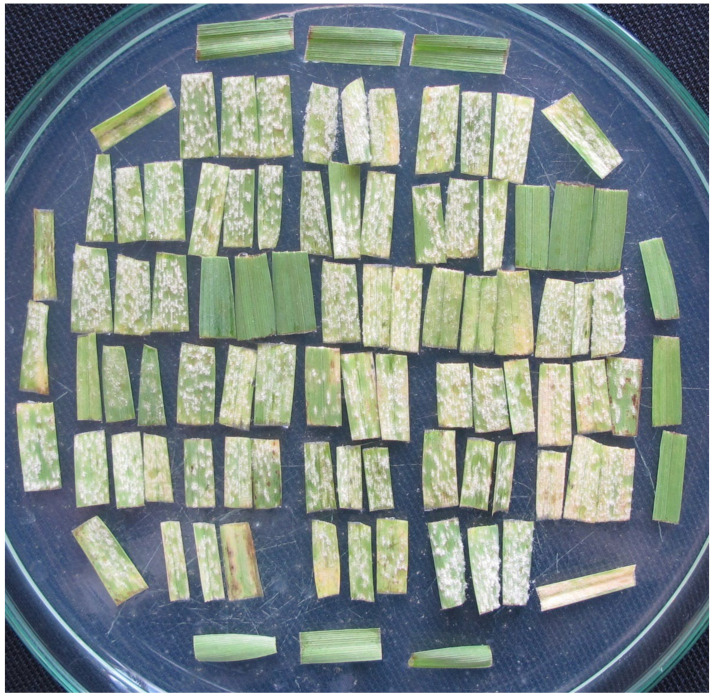
Petri dish with triplets of leaf segments of 30 barley varieties and four diagonally placed segments of a check susceptible variety seven days after inoculation with a powdery mildew isolate.

**Figure 4:**
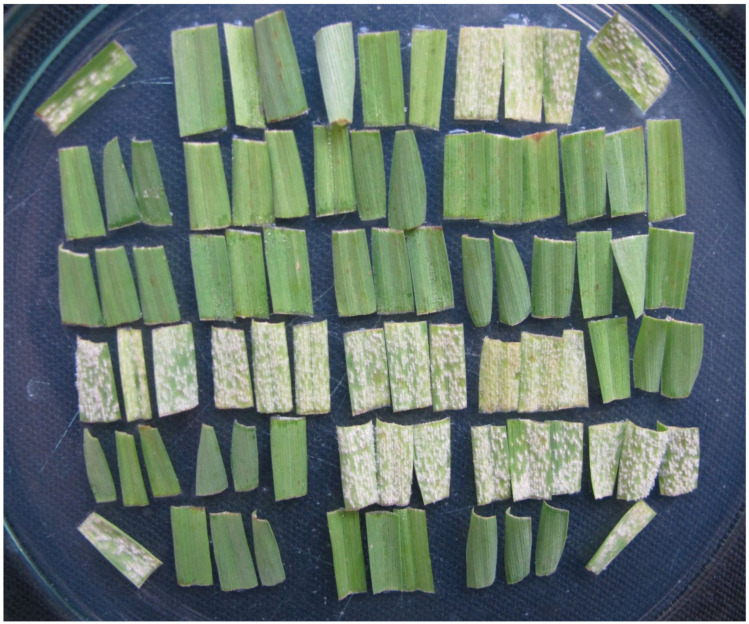
Petri dish with triplets of leaf segments of 26 barley varieties and diagonally placed segments of a check susceptible variety seven days after inoculation with a powdery mildew isolate.

The tests are performed as follows. Usually 30 or sometimes 26 barley varieties are tested weekly and inoculated with 50 to 62 isolates. Each variety is represented by three leaf segments in a Petri dish. Thus, 90 (78) segments of the tested varieties are usually transferred to one dish together with four segments of the susceptible control variety, frequently Stirling or Bowman (Figure 3 and Figure 4).

Each week, the test is divided into two subsets. Mostly on Monday, the segments are cut and placed into the first half of the dishes with agar, and on Tuesday, the dishes with leaf segments are inoculated (one dish with one isolate) with half of the isolates. The other half of the dishes are prepared and inoculated in the same way on Wednesday and Thursday. In one season (from September to the end of June), around 35 weekly tests are carried out. Tests are done to verify different working hypotheses and to facilitate research or breeding requirements.

For tests of accessions in which heterogeneity of resistance was found, samples are individually sown, plant progenies are separately harvested and from each of them (usually five), one segment is tested. In such a case, there are usually 16 pentads on the dishes, i.e., 80 segments, plus four segments of a susceptible control [29]. When testing wheat with narrower leaves than barley, 26 pentads can be placed on the dish [18].

When the complete set of pathogen isolates is not necessary, fewer can be used for inoculation, such as at present when about 2800 spring barley gene bank accessions are screened to detect varieties without genes of specific resistance. In this case, nine isolates with the narrowest virulence spectra are sufficient and eight sets, i.e., 72 dishes with 208 varieties in total, are evaluated every week. The number of isolates used and the design of the tests can vary according to needs.

### 3.3. Phenotyping

The scoring scale to evaluate IRs [28] is supplemented with IR 0(4) (originally 0/(4))—a typical phenotype of the *mlo* gene characterized by the formation of occasional mildew colonies [30]. In our laboratory, we use this expanded IR scale in a modified form IR 0(x), where x can be IRs 2, 3 or 4 and show the occurrence of colonies up to ca. 3% compared to the susceptible control. We also use a similar scale IRs (x) to record a reduced number of colonies ranking from 3 to 30% that occur on varieties carrying particular genes, such as *Ml(Ab),* after inoculation with an avirulent isolate.

IR phenotyping is performed twice, at 7 and 8 dai and significant differences in IRs are re-scored on the same (eighth) day. Special attention is paid to both boundary IRs, i.e., IR 2–3 and IR 3, which represent the greatest risk of error in distinguishing between resistance and susceptibility [31]. If some leaf segments turn yellow, they should be scored one or even two days earlier with a subsequent check of IRs until responses are visible. The results are recorded in a database (Microsoft Excel software) and the IR of one accession–isolate interaction represented with a triplet of leaf segments is usually inserted in a cell including heterogeneous phenotypes, e.g., 4 + 0. In this case, the first digit denotes a more frequent IR, such as two susceptible segments (IR 4) and one fully resistant segment (IR 0). However, when testing progenies of heterogeneous accessions, it is more appropriate to record the phenotypic values of each segment in a separate cell and the scoring of each set of varieties or progenies in one dish is written in one column. After performing the test, one line represents the IRA of one variety or plant progeny.

### 3.4. Postulation of Genes of Specific Resistance

Postulation of genes of specific resistance compares the IRA of the tested variety with previously obtained IRAs of standard varieties carrying, if possible, individual resistance genes (Table 2). If the IRA obtained for the tested variety does not correspond with the IRA of any standard variety, it is classified as an unknown resistance. However, the reliability of this conclusion (i.e., that it is an unknown resistance) depends on how well-equipped a laboratory is regarding (1) its collections of barley standards carrying those genes and (2) pathogen isolates with all known virulence or avirulence and their combinations.

## 4. Conclusions

This description of the method is very detailed, but some points must always be modified, at least with respect to the characteristics of the tested host varieties and isolates of the pathogen.

In our laboratory, this protocol is also used for other instances of phenotyping host–pathogen responses, e.g., for studying the resistance/susceptibility of exotic host varieties, mainly wild barley. Such tests do not aim to postulate resistance genes, but just to characterize any detected resistances in order to obtain their IRAs [16]. Another example is testing wheat (Triticum aestivum L.) with the powdery mildew (Bg tritici) isolates for clustering according to their resistance phenotypes [18].

All cases of postulation are based on the gene-for-gene model [7], where the resistance of host varieties is identified (postulated) on the basis of the known virulence of standard pathogen isolates. Similar tests with identical designs are used to investigate plant pathogen populations, both domestic (central European) [33] and other important parts of the global population [34]. For this purpose, virulence is postulated according to resistance genes of standard host varieties. The method described herein regarding the phenotyping of host–pathogen responses resulting in postulation of genes of specific resistance or virulence can be adapted for performing similar tests on other crops, mainly cereals and their wild relatives [35,36,37,38,39,40]. Collated results have wide applications, as outlined in the introduction.

## 5. Critical Assessment of Leaf Segment Tests Compared to Testing of Whole Plants

Compared to testing of whole plants, the advantages and disadvantages for critical assessment of leaf segment tests are listed below [32,41].

### 5.1. Advantages

- Minimum seed and greenhouse space needed for testing varieties;
- Saving inoculum of isolates and its simple preparation;
- Easy control of the quantity and quality of applied inoculum;
- Leaf segments are oriented horizontally, which allows optimal settling and even distribution of the inoculum;
- Elimination of false heterogeneous responses within variety–isolate interactions resulting from uneven germination and leaf development on pot-grown plants after inoculation;
- In tests of whole plants, the second leaves that often form after inoculation hinder during evaluation;
- Faster, less laborious and more comfortable scoring for the evaluator with easier use of an illuminated lens;
- Rapid finding of variety–isolate interactions that require re-scoring.

### 5.2. Disadvantage

Risk of contamination of the test material in Petri dishes with other fungi (especially of the genus *Mucor*), which causes premature yellowing and rapid decay of leaf segments.

## Figures and Tables

**Table 1:** Scoring scale of nine infection responses recorded on the first leaves of barley varieties after inoculation with a powdery mildew isolate.

Infection Response	Mycelium Growth	Sporulation	Chlorosis/Necrosis
0	None	None	−
0–1	None	None	+
1	Weak	None	+
1–2	Weak	Weak	+
2	Moderate	Weak	+
2–3	Moderate	Moderate	+
3	Strong	Moderate	+
3–4	Strong	Strong	+
4	Strong	Strong	−

Based on Torp et al., 1978 [28].

**Table 2:** Infection response arrays produced by six powdery mildew isolates on five barley genotypes.

Barley	*Ml*	*Blumeria graminis* f. sp. *hordei* Isolates and Year of Their Collection					
Genotype	Resistance	Ch-3-33 ^1^	U-54	I-16	I-167	MNb	E-6
	Gene(s)	2003	2005	2012	2009	2016	2011
Bowman	none	4	4	4	4	4	4
P01	*a1* ^2^	0	0	4	4	0	4
P17	*k1* ^2^	2	4	2	4	4	4
P21	*g* ^2^	0	4	4	0	0	4
Lumar	*a1*, *k1*, *g*^3^	0	0	2	0	0	4

^1^ Responses of isolates to 35 differential genotypes are presented in Dreiseitl 2019 [12]. ^2^ Kølster et al., 1986 [27]. ^3^ Dreiseitl and Jørgensen 2000 [32], Dreiseitl and Zavřelová 2018 [21].

## Data Availability

Not applicable.
